# Assessing COVID-19 susceptibility through analysis of the genetic and epigenetic diversity of ACE2 mediated SARS-CoV-2 entry

**DOI:** 10.2217/pgs-2020-0092

**Published:** 2020-11-27

**Authors:** Georgia Ragia, Vangelis G Manolopoulos

**Affiliations:** ^1^Laboratory of Pharmacology, Medical School, Democritus University of Thrace, Alexandroupolis, 68100, Greece; ^2^Clinical Pharmacology & Pharmacogenetics Unit, Academic General Hospital of Alexandroupolis, Alexandroupolis, 68100, Greece

**Keywords:** (epi)genetics, ACE2, ADAM-17, COVID-19, FURIN, genetic classifier, SARS-CoV-2, TMPRSS2

## Abstract

There is considerable variation in disease course among individuals infected with SARS-CoV-2. Many of them do not exhibit any symptoms, while some others proceed to develop COVID-19; however, severity of COVID-19 symptoms greatly differs among individuals. Focusing on the early events related to SARS-CoV-2 entry to cells through the ACE2 pathway, we describe how variability in (epi)genetic factors can conceivably explain variability in disease course. We specifically focus on variations in *ACE2*, *TMPRSS2* and *FURIN* genes, as central components for SARS-CoV-2 infection, and on other molecules that modulate their expression such as *CALM*, *ADAM-17*, *AR* and *ESRs.* We propose a genetic classifier for predicting SARS-CoV-2 infectivity potential as a preliminary tool for identifying the at-risk-population. This tool can serve as a dynamic scaffold being updated and adapted to validated (epi)genetic data. Overall, the proposed approach holds potential for better personalization of COVID-19 handling.

SARS-CoV-2 emerged in December 2019 as the newest and deadliest member of a family of CoV that invade the respiratory tract of mammals including humans. In many of the humans it infects, SARS-CoV-2 causes mild-to-severe respiratory tract disease which has been named COVID-19. The two previously known members of this family having the ability to infect humans were SARS-CoV and the Middle East respiratory syndrome coronavirus.

There is considerable variation in disease course among individuals infected with SARS-CoV-2. Many of them do not exhibit any symptoms, while some others proceed to develop COVID-19; however, severity of COVID-19 symptoms differs among individuals, and they range from mild, flu-like symptoms, to pneumonia, acute respiratory distress syndrome and even death. In addition, there appears to be sex-related as well as age-related differences [1,2]. Overall, genetic diversity may be related to differences between individuals regarding: infection prognosis, disease severity, response to various types of pharmacological and other forms of therapy, such as oxygen. While efforts are focusing on identifying and developing effective pharmacologic strategies, precision medicine holds promise to substantially aid at unrevealing the epigenetic variability in SARS-CoV-2 infection. Our review focuses on the early events related to SARS-CoV-2 entry to cells. It aims to identify specific genetic and epigenetic markers affecting SARS-CoV-2 entry that can potentially identify individuals who have *a priori* decreased or increased risk for SARS-CoV-2 infection, or who may exhibit reduced or increased potential to establish COVID-19 once infected.

SARS-CoV-2 infects the host by binding to human ACE2. This interaction with ACE2 is mediated via the spike (S) glycoprotein on SARS-CoV-2 surface. During infection, the S protein is cleaved into subunits, S1 and S2. S1 contains the receptor binding domain which allows SARS-CoV-2 to directly bind to the peptidase domain of ACE2. This cleavage is mediated by furin [3]. S2 then likely plays a role in membrane fusion. Subsequent S protein priming relies upon human TMPRSS2 and is essential for entry of SARS-CoV-2. SARS-CoV-2 uses membrane-bound ACE2 as the entry receptor [4,5]. However, the ectodomain of ACE2 can be shed endogenously by ADAM-17 [6]. This soluble form of ACE2 lacks the membrane anchor and circulates in small amounts in the blood. Additionally, CALM has been shown to interact with ACE2 and inhibit shedding of its ectodomain [7]. The ACE2-based-SARS-CoV2 entry machinery to host cells is depicted in Figure 1A.

**Figure 1.**
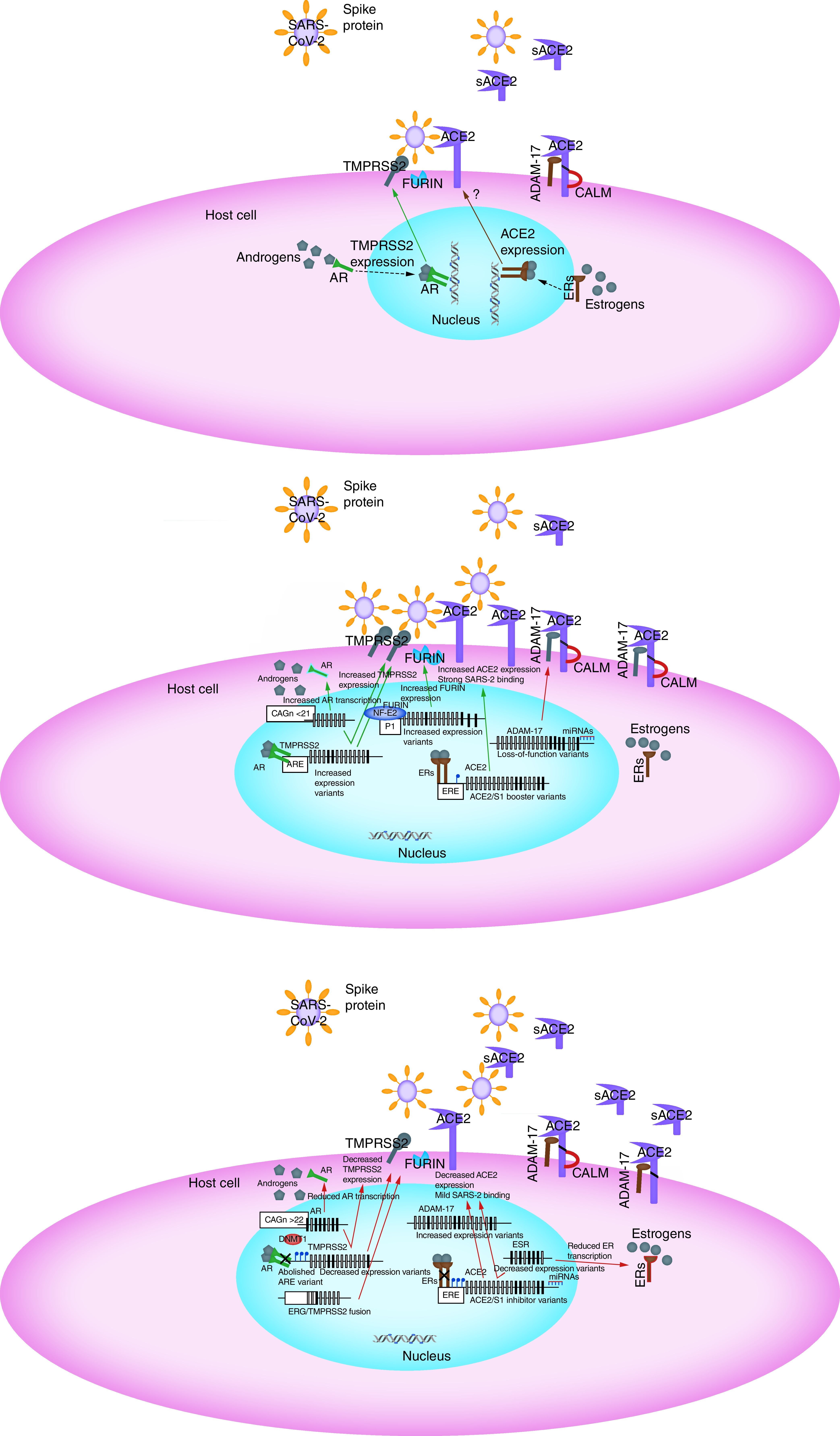
The ACE2-based-severe acute respiratory syndrome coronavirus-2 entry machinery and its regulation by genetic and epigenetic factors. **(A)** SARS-CoV-2 binds to membrane-bound ACE2 to enter the host cells. This interaction with ACE2 is mediated via the spike (S) glycoprotein on SARS-CoV-2 surface. During infection, the S protein is cleaved by furin into subunits, S1 and S2. S protein priming is essential for entry of SARS-CoV-2 and relies upon TMPRSS2. The ectodomain of ACE2 can be shed by ADAM-17 generating sACE2. CALM interacts with ACE2 and inhibits shedding of its ectodomain. AR upregulates TMPRSS2 expression, whereas ERs potentially downregulate ACE2 expression. **(B)** Genetic and epigenetic variations that affect the expression and activity of ACE2-based-SARS-CoV2 entry machinery and strengthen the interaction of SARS-CoV-2 with ACE2 conferring increased infectivity risk. These include: *ACE2* variants that boost the interaction with S protein, decreased *ACE2* methylation that increase ACE2 transcription and available membrane ACE2, *TMPRSS2* increased expression variants that enhance S protein priming, *FURIN* increased expression variants that induce S1/S2 cleavage, *ADAM-17* loss-of-function variants and ADAM-17 downregulation by miRNAs that reduce ADAM-17 mediated ACE2 ectodomain shedding and variations in *AR* gene that increase AR transcription and promote TMPRSS2 expression. **(C)** Genetic and epigenetic variations that affect the expression and activity of ACE2-based-SARS-CoV-2 entry machinery and inhibit the interaction of SARS-CoV-2 with ACE2 conferring reduced infectivity risk. These include *ACE2* variants that inhibit the interaction with S protein, increased *ACE2* methylation and ACE2 downregulation by miRNAs that reduce available membrane ACE2, *TMPRSS2* variants within AR element, *TMPRSS2* fusion with *ERG* and increased *TMPRSS2* methylation that decrease S protein priming, *ADAM-17* increased expression variants that enhance ADAM-17 mediated ACE2 ectodomain shedding and increase sACE2 that binds SARS-CoV-2 thus preventing its entry to host cell, variations in *AR* gene that reduce AR transcription and signaling for TMPRSS2 expression and variations in *ESR* genes that reduce ER transcription and signaling for ACE2 expression. Upregulation is represented by green. Downregulation is represented by red. sACE2: Soluble ACE2; SARS-CoV-2: Severe acute respiratory syndrome coronavirus-2.

It will be interesting to determine whether (epi)genetic variation in loci encoding for molecules that enable SARS-CoV-2 entry in human (namely *ACE2*, *TMPRSS2*, *FURIN*, *ADAM-17* and *CALM*) and in other genetic factors that regulate this ACE2-centered entry machinery for SARS-CoV-2 such as AR and ERs, correlates with altered virus ability to enter the cells and provoke severe disease. Such variation may also contribute to the understanding of gender differences observed in SARS-CoV-2 pandemia. Once (epi)genetic associations are established, they can be used in identifying the at-risk-population that will benefit mostly from early intervention against SARS-CoV-2. Additionally, it may contribute toward developing more personalized approaches to drug therapy of COVID-19 with drugs aimed at the ACE2 pathway. Thus, we scanned the literature to identify polymorphic loci that may contribute to the observed heterogeneity in the incidence and severity of COVID-19 between subjects. In addition, to translate current evidence into a tangible tool, we have attempted to critically assign these variations into a preliminary polygenic risk classifier that discriminates individuals carrying a reduced-infectivity genotypic combination and those who are genetically burdened toward a stronger interaction of SARS-CoV-2 with host cell entry machinery. The way the described genetic and epigenetic variations potentially affect SARS-CoV2 infectivity is depicted in Figure 1B & C and compared with Figure 1A that depicts the reference mechanism.

## Genetic & epigenetic variation in genes involved in SARS-CoV-2 entry

### ACE2

#### *ACE2* genetic variation

ACE2, discovered nearly 20 years ago, is an enzyme that converts angiotensin I to angiotensin 1–9, a peptide of unknown function, and angiotensin II to angiotensin 1–7, a vasodilator. The encoded protein is a functional receptor for the S glycoprotein of the human coronaviruses SARS, HCoV-NL63 and SARS-CoV-2 [8–10]. *ACE2* gene, encoded on minus strand of chromosome Xp22.2 spans 48037 bp and contains 20 introns and 18 exons [11]. Males are hemizygous while females, because of X chromosome inactivation, are a natural mosaic depending on the random process of X chromosome inactivation at early fetus development stages.

In humans, ACE2 is mainly expressed in the cardiovascular, renal and gastrointestinal tissues. Moreover, ACE2 also has been found in the brain, lung and testis [12]. The *ACE2* gene exhibits a high degree of genetic polymorphism with more than 1700 variants identified, including nonsense, missense and intron variants as well as variations in the 3′-UTR. None of the variants has been investigated as for their functional effect. Predicted effects of *ACE2* gene polymorphisms include possible implications on gene splicing, transcription-binding sites and/or ACE2 post-transcriptional regulation. Additionally, genetic variations that affect intermolecular interaction with the SARS-CoV-2 S protein are discussed in terms of virus susceptibility.

Genetic variation in and around the gene encoding *ACE2* is a strong candidate for differences in ACE2 activity. The association of *ACE2* gene polymorphisms with various cardiovascular disease phenotypes (hypertension, left ventricular hypertrophy, coronary artery disease and myocardial infarction), as well as Type 2 diabetes (T2DM) and pulmonary disease, has been the subject of several studies, with conflicting results [13]. Despite the biological proximity of the phenotype of ACE2 activity to genetic variation of the *ACE2* gene, studies assessing the *ACE2* gene variation that leads to changes in circulating ACE2 activity or ACE2 gene expression, transcriptional activity and enzyme levels in tissues or cells are limited (Table 1). The National Center for Biotechnology Information (NCBI) Genotype-Tissue Expression (GTEx) database indicates that the *ACE2* rs879922 polymorphism in intron 11 is associated with the relative expression of ACE2 in normal tibial nerve, with subjects carrying the rs879922 CC genotype having significantly lower ACE2 expression compared with those carrying the GG/CG genotype [14]. It should also be kept in mind that changes of ACE2 expression can be reflected in protein rather than mRNA level as it was shown in mice [15]. For rs2106809, rs4646155 and rs879922 polymorphisms, circulating ACE2 levels did not correlate with genotypes [16]. Finally, the association of the three rs2285666 genotypes with ACE2 protein level measured in serum by ELISA was reported, with the AA genotype having an expression level almost 50% lower than the GG genotype in T2DM patients [17]. At this time, studies on genotype-dependent ACE2 expression in SARS-CoV-2 patients are lacking.

**Table 1.** Genetic and epigenetic variations in *ACE2* and their way to modulate ACE2 protein and/or different virus entry.

*ACE2* gene	Variation, MAF (%)	Effect on protein, virus interaction or infection	Ref.
Genetic variations in exons	rs73635825A >G (S19P), G:0–0.1	Affecting intermolecular interaction with the coronavirus spike protein	[18]
		Interaction-booster between ACE2 and S1	[19]
	rs143936283T >C (E329G), C: <0.02	Affecting intermolecular interaction with the coronavirus spike protein	[18]
	rs778030746T >C (I21V), C:<0.002; rs1244687367A >G (I21T), G: <0.001; rs756231991C >T (E23K), T:<0.001; rs1434130600C >T (A25T), T: <0.001; rs4646116T >C (K26R), C: 2–9;	Interaction-booster between ACE2 and S1	[19]
	rs781255386T >C (T27A), C: 0.001; rs778500138T >A (E35D), A: 0–0.3; rs1199100713A >T (N64K), T: <0.001; rs867318181T >C (E75G), C: <0.001; rs763395248G >A (T92I), A: <0.002; rs1395878099T >G (Q102P), G: <0.004; rs142984500T >C (H378R), C: 0.007–0.02; rs1348114695C >T (E35K), T:<0.002; rs146676783C >T (E37K); T: 0.002–0.003; rs1192192618T >A (Y50F), A: <0.001; rs760159085T >C (N51D), C: <0.001; rs1569243690T >C (N51S), C: <0.001; rs1325542104T >C (M62V), C: <0.02; rs755691167T >C (K68E), C: <0.001; rs1256007252A >C (F72V), C: <0.001; rs766996587C >A, T (M82I), T: 0.001–0.01; rs759579097C >T (G326E), T: <0.001; rs143936283T >C (E329G), C: 0.01–0.03; rs370610075C >A (G352V), A: 0.001–0.01; rs961360700C >T (D355N), T: <0.01; rs751572714T >A (Q388L), 0.002–0.004; rs762890235G >T (P389H), T: 0.002–0.004; rs1016409802T >C (H505R), Unknown; rs1352194082G >A, C (R514G/*), C: <0.001; rs1263424292T >C (Y515C), C: <0.001	Interaction-inhibitor between ACE2 and S1	[19]
Genetic variations in introns	rs2106809A >G (intron 1), G: 18–32	No effect on circulating ACE2 levels	[16]
		No association with SARS poor outcomes	[20]
	rs4646155C >T (intron 8), T: 0–6	No effect on circulating ACE2 levels	[16]
	rs879922G >C (intron 11), C: 32–39	No effect on circulating ACE2 levels	[16]
		Lower ACE2 expression in CC	[14]
	rs2285666G >A (intron 3), A:18	Decreased ACE2 expression in AA	[17]
		No association with SARS poor outcomes	[20]
		No association with SARS process	[21]
	rs4646142G >A, C (intron 7), C: 20–48; rs714205C >G (intron 16), G: 17–49; rs2074192C >T (intron 16), T: 36–47	No association with SARS poor outcomes	[20]
Epigenetic changes: DNA methylation	ChrX: 15621573–15622147	Gender differences in the methylation of specific CpG sites in healthy individuals	[22]
Epigenetic changes: microRNAs	miR-421, miR-143	Are among known ACE2 expression regulators	[23]
	miR-200b, hsa-miR-200c and hsa-miR-429	Predicted miR binding site	[24]
	miR-200c-3p	Mi-R upregulated by avian influenza virus H5N1	[25]
Epigenetic changes: histone modifications	*HAT1, HDAC2* and *KDM5B*	ACE2 regulation in the lung	[26]
	JAK–STAT pathway	*ACE2* gene regulation	[27]

MAF: Minor Allele Frequency; SARS: Severe Acute Respiratory Syndrome.

In a recent study, coding variants of *ACE2* corresponding to the reported binding sites for its attachment with coronavirus S protein were selected and molecular models of these variants were constructed by homology modeling [18]. *ACE2* alleles, rs73635825 (S19P) and rs143936283 (E329G) showed noticeable variations in their intermolecular interactions with the viral S protein. *ACE2* variants that have been associated with ACE2/SARS-CoV-2 interaction are summarized in Table 1. Additionally, ACE2 amino acid residues that interact with SARS-CoV-2 S protein have been described [28]. These modeling observations constitute a promising starting point for further experimental investigations on *ACE2* variants in real clinical setting.

The potential association of *ACE2* gene variations with susceptibility or disease course in patients with previous coronavirus infections (SARS or MERS) has been scarcely studied. Our search has retrieved only two studies. In a 2004 study, frequencies of five out of 103 identified *ACE2* polymorphisms were compared between 168 SARS-CoV patients (among whom 30 males and 16 females had poor outcomes) and 328 healthy volunteers in Hong Kong [20]. No differences in allele frequencies between the different groups were detected and it was concluded that, despite its X-chromosome location, poor outcomes in male SARS patients do not appear to be related to genetic variants of *ACE2*. Similarly, in another case–control study involving 44 SARS cases, 16 anti-SARS-CoV antibody-positive contacts, 87 antibody-negative contacts and 50 non contacts in Vietnam, no evidence emerged that the *ACE2* gene polymorphisms are involved in the disease process in this population. Nevertheless, the authors identified several novel *ACE2* SNPs suggesting that *ACE2* presents with a high variability among populations [21]. However, at present, similar studies in COVID-19 patients are lacking and the abovementioned findings cannot be extrapolated to SARS-CoV-2 for several reasons. At first, just recently, it was shown than key residue substitutions in SARS-CoV-2 C-terminal domain slightly strengthen the interaction with ACE2 and lead to higher affinity for receptor binding than SARS [29]. Additionally, contrary to SARS or MERS, the large population affected by SARS-CoV-2 allows for well-powered genetic studies that can identify both the potential effect of rare *ACE2* variants on SARS-CoV-2 susceptibility or disease course as well as possible differences among different populations. Therefore, the possibility remains that *ACE2* gene polymorphisms may have a significant effect on SARS-CoV-2 infection and COVID-19 symptoms.

The potential effect of *ACE2* extreme rare variants resulting in altered ACE2 expression should also be studied. SARS-CoV-2 resistant individuals should be dully assessed for identifying such rare variants and it should be kept in mind that – if not frequent enough – they cannot be identified in genome-wide association studies. The proportion of such population is expected to be low, however, studying of rare variants can shed light into the functional role of ACE2 expression on SARS-CoV-2 infection. Indeed, Gibson *et al.* have presented a theoretical modeling of rare *ACE2* coding variants documented to occur naturally in several human super- and sub-populations [30]. They showed that rare variants predicted to affect the binding of ACE2 to SARS-CoV-2 S protein do exist in humans.

Toward this direction, three recent papers indicate several nonsense variants that interfere with ACE2-SARS-CoV-2 interaction. Darbani extracted human genetic data from the GenBank, the database for SNPs (dbSNP) including the 1000 genomes project data, the exome aggregation consortium data, and the genome aggregation data, and was able to identify 13 *ACE2* rare missense variants as the interaction-booster between ACE2 and S1, whereas another group of 18 rare SNPs were identified as interaction-inhibitor variants [19]. The author proposes that it is worth investigating the enrichment of the rare variants among the SARS-CoV-2 infected cases with severe symptoms. Another preliminary study (preprint that has not been certified by peer review) gathering genetic data from the Network of Italian Genomes (NIG), described the genetic variation of *ACE2* in the Italian population [31]. The authors identified three common and 30 rare missense variants. Among them, p.Asn720Asp affects a residue located close to the cleavage sequence of TMPRSS2 and likely affects the cleavage-dependent virus intake, whereas p.Trp69Cys, p.Leu351Val and p.Pro389His were predicted to cause conformational changes impacting interaction with ACE2 receptor binding domain. Finally, in a different preprint study comparing more than 200,000 DNA sequences, the authors have identified in *ACE2* gene 11 coding variants in 83 individuals that changed the specific amino acids shown to physically interact with SARS-CoV-2, and an additional 29 variants in 1885 individuals that were within two amino acids of these crucial sites [32].

#### Epigenetic regulation of *ACE2*

Regulation of the *ACE2* gene promoter is poorly understood. ACE2 is transcribed from a proximal and distal promoter. Several enhancer-like sites have been identified downstream, upstream and between *ACE2* exons [33]. Interestingly, among these regulatory regions there are many ER-binding motifs and a few AR-binding motifs. All these sites are potential causes of ACE2 variability among individuals.

The effect of DNA methylation on *ACE2* transcription is even less studied. In the study of Fan *et al.* assessing the effect of *ACE2* DNA methylation on hypertension, a CpG island was identified in the *ACE2* promoter and, subsequently, a fragment containing five CpG dinucleotides in this island was selected and the percentage DNA methylation level was calculated in patients and in healthy individuals [22]. The authors have found gender differences in the methylation of specific CpG sites in healthy individuals with some sites being over-methylated and other under-methylated in males as compared with females (after adjustment for confounding factors). A different pattern of DNA methylation between genders is not uncommon in health and disease [34]. The entry of SARS-CoV-2 into cells through membrane fusion markedly downregulates ACE2 receptors [35]. Whether this downregulation involves *ACE2* DNA methylation alterations needs to be explored.

Additionally, ACE2 expression is inhibited by microRNAs (miRs). MiR-421 and -143 are among known ACE2 expression regulators [23]. In *ACE2* 3′-UTR a predicted miR-binding site has been proposed [24]. The predicted miR-binding site is an exact match to the miRs hsa-miR-200b, hsa-miR-200c and hsa-miR-429. Level of complexity increases since polymorphisms have been identified in all abovementioned miRs. Downregulation of ACE2 is also associated with the acute lung injury or acute respiratory distress syndrome induced by avian influenza virus, SARS-CoV, respiratory syncytial virus and sepsis. For avian influenza virus H5N1 it was shown that it induced the upregulation of miR-200c-3p that targets *ACE2* 3′-UTR [25]. Additionally, it was recently shown that JARID1B, encoded by the *KDM5B* gene, can indirectly affect ACE2/TMPRSS2 expression by repressing transcription of hsa-let-7e/hsa-mir-125a and hsa-mir-141/hsa-miR-200 miRNA families (including miR-141, miR-200a, miR-200b, miR-200c and miR-429) which are targeting these genes [36].

Epigenetic changes that regulate many normal and disease-related processes also include histone modifications. Histones can be modified post-translationally in different ways altering their interactions with DNA and nuclear proteins, thus leading to changes in chromatin architecture and gene activation. Only limited data exist on the potential effect of histone modifications on ACE2 expression. In 2015, Tikoo *et al.* suggested that atorvastatin is associated with increased expression of ACE2 in atherosclerotic rabbits by altering the histone modifications [37]. Systems biology approaches on transcriptome samples from patients with comorbidities associated with severe COVID-19 also propose several potential epigenetic regulators of ACE2 in the human lung, including genes related to histone modifications, such as *HAT1*, *HDAC2* and *KDM5B* [26]. Also, preliminary data of a mouse mammary tissue study implicate the pan JAK–STAT pathway (that affects chromatin structure via histone modifications) in *ACE2* gene regulation [27].

One possibility that has been proposed for SARS-CoV-2 is that variants in the *ACE2* gene for a lung cell receptor could make it easier or harder for the virus to infect these cells; variants that enable viral entry might lead to more extensive lung infection and more serious symptoms, especially since these are the cells that normally produce surfactant, a substance that helps lungs to work properly. Undoubtedly, the role of ACE2 in SARS-CoV-2 infection is critical. Evidence constantly emerges indicating that ACE2 up- or downregulation or conformational alterations (governed by gene polymorphisms, DNA methylation, miRs and polymorphisms within miR encoding genes or histone modifications) may result in altered virus entrance ability into cells. Currently, for *ACE2* the effect of genetic variability on ACE2 expression or function is largely unexplored. As for the role of *ACE2* (epi)genetic variations in COVID-19 course severity, it merits further investigation in real setting patients. It appears that rare nonsense *ACE2* variants are potentially critical players in SARS-CoV-2 binding and entry to host cells. Taking into account that there is a plethora of such variants, *ACE2* exome-sequencing appears as an attractive approach for revealing additional functional *ACE2* variants and identifying those patients who are at increased risk for severe disease as well as those individuals that are (partially) resistant.

### TMPRSS2

#### *TMPRSS2* genetic variation

*TMPRSS2* gene encodes for transmembrane protease serine 2, a serine protease that is essential for viral infectivity. It proteolytically cleaves and activates the S glycoproteins of human coronavirus and the fusion glycoproteins of other viruses such as HMPV and HPIV, and is involved in the proteolytic cleavage and activation of hemagglutinin (HA) protein, making TMPRSS2 essential for spread and pathogenesis of influenza A virus (strains H1N1, H3N2 and H7N9). In SARS-CoV-2 infection, TMPRSS2 is involved in SARS-CoV-2 S protein priming [4].

Several gene polymorphisms have been identified within *TMPRSS2* locus. Their potential association with increased risk of severe COVID-19 course merits further study. Specifically, two *TMPRSS2* intronic variants, rs2070788G >A and rs383510T >C (Table 2), are associated with genotype-specific TMPRSS2 expression in human lung tissues [38]. Individuals with rs2070788GG genotype have the highest expression, GA heterozygotes have intermediate expression and AA homozygotes have the lowest expression of TMPRSS2. Similarly, rs383510T variant exhibits a significantly higher transcriptional level than the C variant [38]. In the same study, rs2070788 and rs383510 variants were significantly associated with higher risk to severe A(H1N1)2009 and A(H7N9) influenza, with individuals carrying the increased *TMPRSS2* expression alleles being at approximately twofold higher risk for severe infection [38].

**Table 2.** Genetic and epigenetic variations in *TMPRSS2* and their way to modulate severe acute respiratory syndrome coronavirus-2 entry to host cell.

*TMPRSS2* gene	Variation	MAF	Effect on protein, virus interaction or infection	Ref.
Genetic variations	rs2070788G >A, intron variant	A: 36–47%	Higher-expression of TMPRSS2 in rs2070788G allele carriers Higher risk to severe A(H1N1)2009 and A(H7N9) influenza	[38]
	rs383510T >C, intron variant	C: 35–49%	Higher TMPRSS2 transcriptional level in rs383510T allele carriers Higher risk to severe A(H1N1)2009 and A(H7N9) influenza	[38]
	rs8134378G >A, T, within androgen response element	A: 0.4–17%	Reduces binding and transactivation by the androgen receptor	[39]
	rs12329760C >T, V160M	T: 15–43%	Significantly associated with fusion by deletion	[40]
Gene fusion	*TMPRSS2*-*ERG* fusion, oncogenic rearrangement	–	Significantly reduced expression of TMPRSS2	[41]
Epigenetic changes: histone modifications	Histone acetylation	–	Associated with promoted prostate cancer cell growth through TMPRSS2 activation	[42]

MAF: Minor Allele Frequency.

Accumulated data reveal a direct role of TMPRSS2 enzyme to SARS-CoV-2 infectivity in males and thus strengthen the idea that *TMPRSS2* genetic variations can impact SARS-CoV-2 severity. To explain the fact that males present with increased COVID-19 symptom severity, a recent study suggested that androgen expression might be related [43]. This is based on the preliminary observation of high frequency of male pattern hair loss among admitted COVID-19 patients. Interestingly, the human *TMPRSS2* gene promoter has a 15-bp androgen response element. The upregulation of *TMPRSS2* mRNA by androgen appears to be mediated by the AR [39], implying that gender differences are expected through SARS-CoV-2 infection. Moreover, within the *TMPRSS2* androgen response element, rs8134378 SNP reduces binding and transactivation by the AR [39]. It could thus be speculated that individuals carrying rs8134378 or other polymorphisms that interfere with this AR-regulated TMPRSS2 stimulation have lower expression of TMPRSS2 and are therefore less vulnerable to SARS-CoV-2 infection.

*TMPRSS2* genetic variations have also been linked with molecular alterations, such as the molecular subtype of *TMPRSS2–ERG* fusion. *TMPRSS2–ERG* fusion is the most common oncogenic rearrangement in prostate cancer. In this chromosomal rearrangement one *TMPRSS2* allele loses its promoter, and one of the *ERG* alleles gains that promoter leading to its overexpression in prostate tumor cells. Results of an *in vivo* study in these cells showed a significantly reduced expression of TMPRSS2 in malignant cells harboring *TMPRSS2–ERG* fusion, but not in prostate cancer cells without *TMPRSS2–ERG* fusion [41]. In studies focused in pancreatic cancer, *TMPRSS2* rs12329760 allele was associated with fusion by deletion [40,44]. Evidence also suggests that men with *TMPRSS2:ERG* positive tumors may have longer prostate cancer survival after androgen-deprivation therapy (ADT) and this is further discussed in the section of *AR* gene polymorphisms. Based on these findings, it can be hypothesized that *TMPRSS2-ERG* fusion positive prostate cancer patients are less vulnerable to SARS-CoV-2 infection. Some supporting – albeit circumstantial – evidence in this direction, comes from a study by a group in Italy containing data from 9280 subjects (4532 males) with laboratory-confirmed SARS-CoV-2 infection from 68 hospitals in the area of Veneto [45]. They calculated that in the Veneto male population (2.4 Million men), 0.2% and 0.3% of non-cancer and cancer patients, respectively, tested positive for SARS-CoV-2. They concluded that cancer patients overall have an increased risk of SARS-CoV-2 infections than non-cancer patients, however, details on *TMPRSS2:ERG* patient status are missing. Collectively, this information implies that *TMPRSS2* genetic variations or gene rearrangement potentially affect, in different ways, SARS-CoV-2 susceptibility, symptom manifestation or virus pleiotropy.

#### Epigenetic regulation of *TMPRSS2*

Hardly any studies have examined whether DNA methylation is involved in the regulation of *TMPRSS2*. However, prostate cancer is tightly controlled by epigenetic regulation [46]. Evidence from AR-negative prostate cancer cells shows that DNMT1 is associated with hypermethylation of *TMPRSS2* gene and low expression level of TMPRSS2 [47]. Additionally, it has been reported that epigenetic events affected by genetic variation differentially regulate miRs in African American prostate cancer patients and are drivers of *TMPRSS2:ERG*-negative tumors [48]. Histone acetylation has also been proposed to be associated with promoted prostate cancer cell growth [42]. Hopefully soon epidemiologic analysis of data from prostate cancer patients will show whether they exhibit a decreased rate of SARS-CoV-2 infection.

### Furin

Furin is a cellular endoprotease that catalyzes the proteolytic activation of proprotein substrates in the secretory pathway compartments [49]. Additionally, furin has an emerging role in virology, since many pathogenic viruses, such as avian influenza virus, HIV-1, measles virus and RSV, express envelope glycoproteins that must be cleaved at consensus furin sites to form the mature and fusogenic envelope glycoprotein [50]. Just recently, it was shown that SARS-CoV-2 S glycoprotein contains a potential cleavage site for furin proteases. Hoffmann *et al.* have studied the contribution of this multibasic cleavage site to SARS-CoV-2 infection of human cells and showed that SARS-CoV-2 depends on furin-mediated precleavage of its S protein at the S1/S2 site for subsequent S protein activation by TMPRSS2 in lung cells [3].

The human *FURIN* gene, located on chromosome 15q26.1 consists of 16 exons and 15 introns that encode 795 amino acid residues. Three promoters (P1, P1A and P1B), each harboring an alternative furin transcription start site, have been described, however, they are predicted to express the same protein. Interestingly, the P1 promoter binds the transcription factor C/EBPβ and can be *trans*-activated upon cytokine stimulation [50]. Several gene polymorphisms have been identified in *FURIN* gene (Table 3). Among them, rs17514846, that leads to higher furin expression in vascular endothelial cells [51], was associated with the prevalence of metabolic syndrome [52]. More intriguingly, a SNP in the P1 promoter of the *FURIN* gene, rs4932178C >T, has been associated with increased risk of developing persistent HBV infection with detectable amounts of HBeAg in the serum [53]. rs4932178T allele increases the binding efficiency of the hepatic transcription factor NF-E2, leading to approximately threefold increase in the transcriptional activity of the allele T promoter. Patients with persistent infection were significantly more likely to carry allele T and less likely to carry allele C [53]. It is well known that furin plays a key role in processing of HBeAg precursor that is essential for the development of chronic HBV infection, into maturated HBeAg. This way, *FURIN* rs4932178C >T is an attractive candidate for SARS-CoV-2 infection potentially identifying individuals with increased furin transcription and therefore at risk for virus entry.

**Table 3.** Genetic variations in *FURIN* and their way to modulate severe acute respiratory syndrome coronavirus-2 entry to host cell.

*FURIN* gene	Variation	MAF	Effect on protein, virus interaction or infection	Ref.
Genetic variations	rs4932178C >T	T: 14–40%	Approximately threefold increase in the transcriptional activity of the allele T promoter Increased risk of developing persistent HBV infection	[53]
	rs17514846C >A, G, T	A: 14–49%	Increased furin expression in vascular endothelial cells	[51]

HBV: Hepatitis B Virus; MAF: Minor Allele Frequency.

### ADAM-17

The ADAMs is a family of transmembrane and secreted proteins implicated in a variety of cellular processes, including processing of proteins, interactions with integrin receptors and with signaling molecules. ADAM-17 is widely expressed in various tissues including bronchial epithelial cells, vascular smooth muscle cells and macrophages in the lung. ADAM-17 has a central role in inflammation, ischemic stroke, memory, brain repair, neuroinflammatory disorders, malignancies, heart diseases, atherosclerosis, diabetes kidney disease and other [54]. The most well-established function ADAM-17 is to cleave ectodomains of various transmembrane proteins including the ectodomain of ACE2. For ACE/furin interaction, the ACE2 cleavage site has been identified [55]. Upregulation of ACE2 shedding could modulate high levels of shed ACE2 [6], leading to inhibition of SARS-CoV-2 infectivity in the presence of a competition between ADAM-17 and TMPRSS2 for ACE2 processing.

*ADAM-17* genomic DNA extends 66505 base pairs with 19 exons at cytogenetic location 2p25.1. Several genetic variations of *ADAM-17* were shown to be involved in various inflammation-related diseases. Among *ADAM-17* identified gene polymorphisms, C-154A, Ser747Leu (rs55796712G >A), −25T/G and rs12692386A >G promoter polymorphism, contribute to ADAM-17 expression upregulation (Table 4) [56,57]. It is tempting to speculate that *ADAM-17* gene polymorphisms associated with increased ADAM-17 levels and activity can be associated with enhanced shedding and increase of sACE2 levels interfering this way with SARS-CoV-2 entry into cells. Rare non synonymous variants have also been identified in *ADAM-17*. Such a variation is rs142946965 (R215I) leading to loss-of-function of ADAM-17 alpha-secretase [58]. On the other hand, *ACE2* mutations can also affect ACE2-ADAM-17 interaction. Interestingly, a point mutation in the ACE2 ectodomain, L584A, markedly attenuated shedding. The resultant ACE2-L584A mutant trafficked to the cell membrane and facilitated SARS-CoV entry into target cells, suggesting that the ACE2 ectodomain regulates its release and that residue L584 might be part of a putative sheddase ‘recognition motif’ [54].

**Table 4.** Genetic variations in *ADAM-17* affecting enzyme expression that potentially modulate severe acute respiratory syndrome coronavirus-2 entry to host cell.

*ADAM-17* gene	Variation	MAF	Effect on protein, virus interaction or infection	Ref.
Genetic variations	rs12692386A >G	G: 18–19%	Increased ADAM-17 mRNA in tissues from abdominal aortic aneurysm patients	[57]
	C-154A		-154A allele was found associated with increased ADAM-17 activity and a 14% increase of sTNF	[56]
	rs142946965C >A, R215I	A: 0.01–0.02%	Loss-of-function of ADAM-17 alpha-secretase	[58]
Epigenetic changes: microRNAs	miR-145		Negative regulator of ADAM-17 expression	[59,60]

MAF: Minor Allele Frequency.

It has been reported that in human non-small-cell lung cancer estradiol enhances ADAM-17 expression and protein levels [61]. Independently or jointly with *ADAM-17* genetic variations this finding would suggest higher ACE2 shedding in female, partially explaining the gender differences so far found in COVID-19 severity course.

Methylation studies on ADAM proteins identified ADAM-12 as the only member of the ADAM family showing noteworthy methylation changes [62]. ADAM-17 expression is subjected to epigenetic regulation via miRs. Specifically, several reports implicate miR-145 as a negative regulator of ADAM-17 expression [59,60]. Overall, the epigenetic component of ADAM-17 regulation remains to be investigated.

### Calmodulin

Calmodulin is an intracellular calcium-binding protein which mediates the Ca^2+^ regulation of a wide range of physiological processes throughout eukaryotic organisms including the regulation of ACE2 ectodomain shedding. Seminal data from computational analysis of the cytoplasmic domain of ACE2 revealed a conserved consensus calmodulin-binding motif. Studies using immunoprecipitation experiments revealed that calmodulin associates with ACE2 suggesting that this motif may be functional [7]. A year later, Lai *et al.* showed that calmodulin binds a 16-amino acid synthetic peptide within the cytoplasmic domain of human ACE2, forming a calcium-dependent calmodulin-peptide complex and, additionally, they provided evidence from human cells (Huh-7 hepatocarcinoma cell line) that the calmodulin-specific inhibitor-stimulated shedding of ACE2 is independent from phorbol ester-induced shedding [63].

In humans, calmodulin is encoded by multiple genes; *CALM1*, *CALM2* and *CALM3* which are found on chromosomes 14q32.11, 2p21 and 19q13.32, respectively. Calmodulin is highly conserved across species with all vertebrate *CALM* genes encoding identical proteins. Given this degree of conservation, it was long thought that mutations in calmodulin were incompatible with life. Nevertheless, to date, several mutations have been identified in human *CALM* genes (~36 in *CALM1*, 23 in *CALM2* and 15 in *CALM3*), some of them associated with life-threatening conditions in childhood and mainly arrhythmias, such as catecholaminergic polymorphic ventricular tachycardia, long QT syndrome and idiopathic ventricular fibrillation [64–66].

It could be speculated that any mutations in *ACE2* altering the residues binding to calmodulin can alter ACE2 ectodomain shedding. On the other hand, it is still unknown whether mutations in any *CALM* gene can modulate ACE2/calmodulin interaction.

### Androgen receptor

AR, a ligand-dependent nuclear transcription factor, binds androgens to exert their biological actions. ARs have an important role in the regulation of TMPRSS2 expression. We have already discussed how *TMPRSS2* gene polymorphisms that reduce binding and transactivation by the AR could potentially reduce SARS-CoV-2 infectivity. Herein, we discuss the potential association of *AR* polymorphisms with TMPRSS2 expression, suggesting their possible suitability as genetic markers for COVID-19 precision medicine.

The *AR* gene, located on the X chromosome at Xq11–12, is more than 90 kb long and codes for a protein that has three major functional domains: the N-terminal domain, DNA-binding domain and androgen-binding domain. Genetic aberrations of the *AR* caused by mutations, rearrangements and polymorphisms result in a mutant receptor that has varied functions compared with wild-type AR [67]. To date, over 1000 mutations have been reported in the *AR* with most of these being associated with androgen insensitivity syndrome. The human *AR* gene contains two polymorphic sites in exon 1 that encodes the entire N-terminal domain (Table 5). They are characterized by different numbers of CAG and GGC repeats resulting in variable lengths of polyglutamine (polyGln/polyQ) and polyglycine (polyGly/polyG) repeat sequences [68]. The normal range of the CAG repeat is 11–31 triplets in length, and the transactivational activity of the AR is inversely associated with the number of CAG repeats; longer CAG repeats result in reduced AR transcriptional activity [69]. The functional consequences of GGC repeat are less clear.

**Table 5.** Genetic variations in *AR* affecting receptor expression that potentially modulate severe acute respiratory syndrome coronavirus-2 entry to host cell.

*AR* gene	Variation	Short tandem repeat range, frequency	Effect on protein, virus interaction or infection	Ref.
Genetic variations	CAG repeats, exon 1, variable lengths of polyglutamine (polyGln/polyQ)	6–35, >21: 13–55%	Longer CAG repeats result in reduced AR transcriptional activity	[69]
	GGC repeats, exon 1, variable lengths of polyglycine (polyGly/polyG)	>17: 1–8%	Longer GGC repeats potentially result in reduced AR transcriptional activity	[69]
Epigenetic changes: histone modifications	Histone demethylases	–	Generation of constitutively active forms of androgen receptor variants	[70]

*AR* mutations and polymorphisms have been extensively studied in prostate cancer and infertility. We will focus on the specific effect of the functional CAGn *AR* polymorphism on TMPRSS2 expression, since TMPRSS2 is expressed in an androgen-dependent manner. Individuals with a lower number of CAG repeats exhibit higher *AR* gene expression levels and generate more functional ARs increasing their sensitivity to androgens. In these individuals, increased TMPRSS2 expression is expected and thereby increased SARS-CoV-2 S priming and virus entry can be speculated. On the other hand, individuals carrying longer CAG repeats have decreased sensitivity to androgens, decreased TMPRSS2 expression and are potentially protected from SARS-CoV-2 infection. The latter is further supported by the recent work of Montopoli *et al.* who provide evidence for the potential use of antiandrogens against SARS-CoV-2 [45]. Comparing the total number of SARS-CoV-2 positive cases, prostate cancer patients receiving ADT had a significantly lower risk of SARS-CoV-2 infection compared with patients who did not receive ADT (OR 4.05; 95% CI: 1.55–10.59). The authors concluded that prostate cancer patients receiving ADT appear to be partially protected from SARS-CoV-2 infections [45]. Cancer systems biology analysis also highlights the role of histone demethylases on the generation of constitutively active forms of AR variants associated with progression of prostate cancer [70–72]. Whether there is a role of AR epigenetics on TMPRSS2 expression and SARS-CoV-2 infectivity needs to be further addressed.

Additionally to *TMPRSS2*, a few AR-binding motifs are among *ACE2* regulatory regions. It is still not known whether these elements are actually active and thus leading to an androgen-dependent increase in responsiveness of the human *ACE2* promoter. If this were true, individuals with a lower number of CAG repeats could also have increased ACE2 expression.

### Estrogen receptors

ERs include ERα and ERβ, encoded by two distinct genes, *ESR1* (6q24–27) and *ESR2* (14q22–24), respectively. ERs have a central role in the mechanism that estrogens induce cellular changes [73]. Estrogens diffuse into the cell and bind to the ERs, located in the nucleus. This nuclear estrogen-ER complex binds to estrogen response element sequences in the promoter region of estrogen-responsive genes, resulting in recruitment of coregulatory proteins (coactivators or corepressors) to the promoter, increased or decreased mRNA levels and associated protein production, and a physiological response [74].

Among *ACE2* regulatory regions, there are many ER-binding motifs. Through binding to ERα, estradiol significantly increased ACE2 expression in human atrial myocardium [75], whereas ACE2 expression was downregulated in kidneys and no effect was found in the lung [76]. Overall, no clear picture is emerging and the role (if any) of estrogen in COVID-19 remains to be elucidated. One study went to the opposite direction hypothesizing that ER activation with conjugated estrogens may be a good prevention and therapeutic strategy against COVID-19, on the basis of findings in animal experiments showing that estrogen treatment silences the inflammatory reactions and decreases virus titers leading to improved survival rate [77]. It is, however, still unclear whether the putative ER-binding motifs from the human *ACE2* promoters have the ability to bind ERs [78].

Different polymorphisms have been described in both the *ESR1* and *ESR2* genes (Table 6). For *ESR1*, the two most studied SNPs are *Pvu*II (397T >C, rs2234693) and *Xba*I (351G >A, rs9340799), both located in intron 1, separated by 46 bp, and often studied as haplotypes [79]. Another popular *ESR1* polymorphism consists of a dinucleotide (TA) repeat upstream of exon 1. The number of repeats ranges between 9 and 27, with frequency peaks at 14 and 23 repeats [80]. The functional effect of these SNPs on ERα has not been elucidated. It has been assumed, however, that both polymorphisms may be in linkage disequilibrium with other unknown variants in the gene, which may affect the gene expression or function, or as intronic changes they may have an impact on the expression of ERα by influencing the transcription through alternative splicing of the mRNA transcript [81].

**Table 6.** Genetic variations in *ESRs* that potentially modulate severe acute respiratory syndrome coronavirus-2 entry to host cell.

Gene	Variation	MAF	Effect on protein, virus interaction or infection	Ref.
*ESR1*	rs2234693T >C, G (*Pvu*II, 397T >C), intron 1	C: 27–48%	May affect the gene expression or function, or as intronic changes they may have an impact on the expression of ERα by influencing the transcription through alternative splicing of the mRNA transcript	[81]
	rs9340799A >G (*Xba*I, 351G >A), intron 1	G: 17–37%	May affect the gene expression or function, or as intronic changes they may have an impact on the expression of ERα by influencing the transcription through alternative splicing of the mRNA transcript	[81]
	dinucleotide (TA) repeat upstream of exon 1		May affect the gene expression or function, or as intronic changes they may have an impact on the expression of ERα by influencing the transcription through alternative splicing of the mRNA transcript	[81]
*ESR2*	rs1256049C >T (*Rsa*I, 1082G >A)	T: 1–8%	Unknown functional significance	
	rs4986938C >T (*Alu*I, 1730G >A)	T: 8–37%	Unknown functional significance	
	nt809(del21)		Results in the deletion of seven amino acids from the D domain of the ERβ protein	
	rare non synonymous 846G >A, exon 4		Unknown functional significance	
	rare synonymous 1421T >C, exon 7		Unknown functional significance	
Epigenetic changes: DNA methylation	DNA hypermethylation in the two tissue-dependent and differentially methylated regions		Expression of *ESR1* is suppressed	[82]
	DNA methylation of the promoter region		Regulates the expression of *ESR2*	[82]
Epigenetic changes: histone modifications	Post-translational histone modifications		Modified estrogen signaling	[83]

MAF: Minor Allele Frequency.

As for *ESR2*, two silent G-to-A SNPs, *Rsa*I (1082G >A, rs1256049) and *Alu*I (1730G >A, rs4986938), have been extensively investigated. Other polymorphisms identified in *ESR2* gene include the nt809(del21) polymorphism, which results in the deletion of seven amino acids from the D domain of the ERβ protein, an exon 4 rare non synonymous change (846G >A) and a rare synonymous 1421T >C transition in exon 7. Their functional significance remains unknown.

Additionally, *ESR1* and *ESR2* are downregulated by DNA methylation. The effect of DNA methylation on *ESR1* expression has been extensively studied in breast cancer. It is well known that tissue-specific expression of *ESR1* in normal tissues is regulated by DNA methylation of two tissue-dependent and differentially methylated regions (T-DMR) located upstream and not within the promoter region of *ESR1* [82]. In the tissues with DNA hypermethylation in the two T-DMRs, the expression of *ESR1* is suppressed. On the other hand, DNA methylation of the promoter region (not the T-DMRs) was reported to regulate the expression of *ESR2*. Estrogen signaling is also tightly connected with post-translational histone modifications whereas several estrogen signaling co-regulators exhibit chromatin-modifying activities [83].

*ESR1* and *ESR2* polymorphisms have been associated with several pathologic conditions such as breast and prostate cancer, osteoporosis, Alzheimer’s disease and cardiovascular diseases [73]. The potential association of genetic and epigenetic alterations on *ESR1* and *ESR2* with SARS-CoV-2 infection can only be hypothesized at present. To test this hypothesis, the role of estrogen in ACE2 expression has to be studied. Once there is a role for estrogen, then the functional effect of *ESR1* and *ESR2* gene polymorphisms needs to be addressed. In the meantime, epidemiological studies on the rate of SARS-CoV-2 infection of breast or ovarian cancer patients treated or not with endocrine therapy can cast a light on the relation of ERs and COVID-19 disease and therapeutics.

## Genetic classifier for SARS-CoV-2 risk group stratification

Polygenic risk score is an approach to calculate the genetic risk to develop a multigenic disease conferred by multiple genetic variants conferring low, moderate or high risk of developing a disease [84]. Currently, attempts to apply a computational algorithm that combines information from all relevant variants into an absolute number (polygenic risk score) depicting the genetic risk for developing a disease have been focused on several multifactorial diseases such as coronary artery disease [85,86], psychiatric diseases [87], T2DM [88] and breast cancer [89]. Polygenic risk score thus reflects the benefits of early detection and treatment of these diseases.

It can be predicted that a polygenic risk score for SARS-CoV-2 infectivity and COVID-19 disease severity will eventually be created. Thus, together with all other environmental factors, genetics may have a significant role and we anticipate that several already known genetic loci will be verified, including the ones described herein, and possibly also new loci will be identified through GWAS and will be associated with the individual variability in virus vulnerability and severity to COVID-19 symptoms. Tables 1–6 summarize the genetic variations in *ACE2*-based SARS-CoV-2 entry machinery pathway, we have discussed in this review that potentially affect SARS-CoV-2 entry to host cells. Based on current evidence, we have attempted to critically assign these variations into a polygenic risk classifier. We therefore propose a preliminary SARS-CoV-2 polygenic risk classifier that discriminates between individuals carrying a reduced-infectivity genotypic combination and those who are genetically burdened toward a stronger interaction of SARS-CoV-2 with host cell entry machinery (Table 7). The impact of the genetic classifier on SARS-CoV-2 attachment to host cells is depicted in Figure 1B & C.

**Table 7.** Genetic variations included in the preliminary severe acute respiratory syndrome coronavirus-2 polygenic risk classifier discriminating individuals carrying low or high infectivity risk.

Type of genetic classifier	Gene	Variations
Variations stratifying individuals in low infectivity risk	*ACE2*	Nonsense variants that abolish virus binding (listed in Table 1)
	*TMPRSS2*	rs8134378
		rs12329760
	*ADAM-17*	C-154A
		Ser747Leu
		-25T/G
		rs12692386
	*AR*	>22 CAG repeats
Variations stratifying individuals in high infectivity risk	*ACE2*	Nonsense variants that enhance virus binding (listed in Table 1)
	*TMPRSS2*	rs2070788
		rs383510
	*FURIN*	rs4932178
	*AR*	≤22 CAG repeats

We suggest that *ACE2* variations should be a core element of this polygenic risk classifier. Current evidence points to a set of *ACE2* nonsense variations that can abolish virus binding and are thus ‘protecting’ from infection, and also to a different set of nonsense variations that enhance virus binding and are therefore ‘predisposing’ to increased infection. *TMPRSS2*, *FURIN*, *ADAM-17* and *AR* gene polymorphisms may also have a role in the SARS-CoV-2 infectivity. In *TMPRSS2*, rs8134378 and rs12329760 are associated with reduced TMPRSS2 expression and can act as protective variations, whereas rs2070788 and rs383510 lead to higher TMPRSS2 expression and are therefore considered in our classifier as predisposing variations. In *FURIN*, rs4932178 variation leads to increased enzyme expression and can also be considered as a predisposing variation. In *ADAM-17*, the variations C-154A, Ser747Leu, −25T/G and rs12692386A >G that contribute to ADAM-17 expression upregulation have been included in the protective classifier. Last but not least, CAG repeat variation in *AR* gene has a place in our classifier. Setting a cutoff for repeats at 22, individuals carrying >22 CAG repeats are at reduced risk due to decreased sensitivity to androgens and thus reduced TMPRSS2 expression and individuals carrying ≤22 are at increased risk due to increased sensitivity to androgens and thus increased TMPRSS2 expression.

## Initiatives on SARS-CoV-2 genetics

Genetic diversity and evolution of SARS-CoV-2 is already subjected to intense analysis. SARS-CoV-2 sequences are currently available in GenBank and the sequence read archive and are updated as additional sequences are released. So far, many mutations and deletions on coding and non coding regions of SARS-CoV-2 have been found [90]. Sequencing SARS-CoV-2 is of great importance since mutation, especially in the spike surface glycoprotein, might induce its conformational changes, which probably lead to antigenicity changing.

In addition to the genetic diversity of the virus, it is of paramount importance to characterize the genetic diversity of the host. Our approach in this review is to identify hypothesis-testing genetic markers that can provide clues as to the heterogeneity of individuals in relation to their propensity to be infected by SAR-CoV-2. However, several efforts are underway aiming to identify individuals at unusually high or low risk using GWAS as well as genome sequencing and other high-throughput approaches. They include:

- The COVID-19 host genetics initiative aiming, among other, to organize analytical activities across studies to identify genetic determinants of COVID-19 susceptibility and severity and to pull individual-level genetic and clinical data together to advance analysis beyond simple GWAS [91];
- Genomics England, in partnership with the GenOMICC (Genetics of Mortality in Critical Care) consortium, has started another initiative in UK aiming to analyze the genomes of COVID-19 patients to try to understand how genes may affect individual reaction to the virus [92]. The study aims to deliver whole genome sequencing of up to 20,000 people who have been severely affected by COVID-19 – requiring intensive care – and 15,000 people who had mild symptoms.

## Conclusion & future perspective

Day by day our understanding of the cell mechanisms SARS-CoV-2 manipulates to establish COVID-19 disease increases. The ACE2-based pathway for SARS-CoV-2 entry to host cells consists a crucial element of this machinery and is quite well characterized. Precision medicine approaches may contribute in advancing our understanding of how to use pathogen and human genomics in public health approaches to prevent and control COVID-19. Genetic and epigenetic variations in *ACE2*, *TMPRSS2* and *FURIN* genes, as central components for SARS-CoV-2 cell entry, and also on other molecules that modulate their expression such as *CALM*, *ADAM-17*, *AR* and *ESRs* can potentially discriminate individuals who are at increased risk for SARS-CoV-2 infection or are potentially resistant. Several polymorphisms have been identified that may affect the activity of the above enzymes. The most promising of them have been combined to generate a preliminary polygenic risk classifier. This is meant more as a paradigm setting exercise and it is expected that soon, with the application of next generation sequencing approaches, more relevant polymorphisms will be identified and a more complete polygenic risk score will be possible. In combination with big data and artificial intelligence, such a tool could be used to predict infection risk in healthy individuals as well as clinical outcomes and possible need for specific types of drug therapy in COVID-19 patients.

The conclusions of this review are largely based on the prevailing assumption that the harming effect of ACE2 is proportional to its expression at the host cells. However, recently a novel hypothesis has been put forward, suggesting that the opposite may actually be the case [93]. We should also acknowledge that in the case of *ACE2*, genetic variability is high and largely unexplored. We expect, however, that intensive ongoing research will soon clarify the effect of relevant functional variants. Therefore, the preliminary tool described herein and any future tool based on genetic analysis of the ACE2-based SARS-CoV-2 entry pathway should be adapted according to ongoing evidence. This approach provides a dynamic scaffold based on current knowledge that can be constantly updated and adapted as novel, validated genetic and epigenetic data keep deriving. Currently, the proposed polygenic character of the risk implies that patient’s characterization would require medium- to high-throughput molecular platform. Indeed, at least for *ACE2*, exome sequencing is herein proposed. Though with current data, it is hard to image cost–effectiveness ratio, the growing scale of human genetics studies, the constant reduction in their costs, and the increasing number of clinical applications for genome sequencing will inevitably lead to a much improved cost–effectiveness ratio in the near future.

Once the risk for SARS-CoV-2 susceptibility has been detected, several strategies can be adapted to mitigate the risk, including societal and clinical interventions. Individuals who are genetically burdened toward a stronger interaction of SARS-CoV-2 with host cell entry machinery will need reinforced precautions such as major social distancing and intensive use of protective measures such as face masks and good personal hygiene to avoid contact with the virus. In the case of infected individuals bearing these more vulnerable variants, early pharmacological intervention or even more aggressive treatment from initial presentation to prevent progression toward worsening of COVID-19 and unfavorable outcomes should be considered.

The present manuscript focuses on genetic markers that can provide clues as to the heterogeneity of individuals in relation to their propensity to be infected by SAR-CoV-2. It should be kept in mind, however, that several of these genetic markers may be useful for pharmacogenetically driven personalization of pharmacological treatment of the disease. As we have recently described [94], several agents that target the virus entry machinery into host cells and consist mainly of ACE2 and TMPRSS2, as well as other cellular molecules regulating ACE2 expression, such as ADAM-17 and calmodulin, have potential for prophylactic and therapeutic intervention at the early stages of SARS-CoV-2 infection and COVID-19 disease. It can be therefore speculated that the described variants affecting ACE2-based pathway for SARS-CoV-2 entry would also have a role in the personalization of treatment with such agents.

Overall, we are confident that personalized medicine tools on COVID-19 will be generated and will become increasingly efficient and precise, allowing for better understanding of the heterogeneity of infection and disease dynamics and facilitating the development and clinical implementation of tailored drugs/drug schemes adapted to the molecular profile and the specific needs of individual patients.

**Executive summary**

**Severe acute respiratory syndrome coronavirus-2 infection mechanism**

- SARS-CoV-2 infects the host by binding to human ACE2 via the spike (S) glycoprotein on SARS-CoV-2 surface.
- The S protein is cleaved by furin into subunits, S1 and S2.
- Subsequent S protein priming relies upon human transmembrane protease, serine 2 (TMPRSS2).
- The ectodomain of ACE2 can be shed endogenously by the disintegrin metalloproteinase 17 (ADAM-17).
- Precision medicine holds promise to substantially aid at unrevealing the (epi)genetic variability in SARS-CoV-2 infection.

**Genetic & epigenetic variation in components enabling SARS-CoV-2 entry**

**ACE2**

- The role of ACE2 in SARS-CoV-2 infection is critical.
- The *ACE2* gene exhibits a high degree of genetic polymorphism.
- *ACE2* rare missense variants were identified as interaction-booster or interaction-inhibitor between ACE2 and S1.
- *ACE2* exome sequencing appears as an attractive approach for identifying those patients who are at increased risk for severe disease as well as identifying the individuals that are (partially) resistant.
- ACE2 expression is regulated by DNA methylation, microRNAs and histone modifications.

**TMPRSS2**

- Several gene polymorphisms have been identified within *TMPRSS2* locus.
- rs2070788G >A and rs383510T >C are associated with genotype-specific TMPRSS2 expression in human lung tissues.
- The upregulation of *TMPRSS2* mRNA by androgen appears to be mediated by the AR.
- Within the *TMPRSS2* androgen response element, a SNP (rs8134378) reduces binding and transactivation by the AR.
- In *TMPRSS2-ERG* fusion, one *TMPRSS2* allele loses its promoter leading to reduced TMPRSS2 expression.
- DNMT1 is associated with hypermethylation of *TMPRSS2* gene and low expression level of TMPRSS2.
- Histone acetylation proposed to be associated with promoted prostate cancer cell growth may also have a role on TMPRSS2 expression.

**Furin**

- Several gene polymorphisms have been identified in *FURIN* gene.
- rs17514846 leads to higher furin expression in vascular endothelial cells.
- rs4932178C >T located in *FURIN* gene promoter leads to approximately threefold increase in the transcriptional activity of the allele T promoter.

**ADAM-17**

- Among *ADAM-17* identified gene polymorphisms, C-154A, Ser747Leu (rs55796712G >A), −25T/G and rs12692386A >G promoter polymorphism, contribute to ADAM-17 expression up-regulation.
- *ADAM-17* gene polymorphisms associated with increased ADAM-17 levels and activity can be associated with enhanced shedding and increase of sACE2 levels interfering this way with SARS-CoV-2 entry into cells.

**Calmodulin**

- CALM prevents ACE2 ectodomain shedding.
- Several mutations have been identified in human *CALM* genes.
- It is still unknown whether mutations in any *CALM* gene can modulate ACE2/CALM interaction.

**Androgen receptor**

- ARs have an important role in the regulation of TMPRSS2 expression.
- CAGn *AR* polymorphism affects TMPRSS2 expression.
- Lower number of CAG repeats increase TMPRSS2 expression, longer CAG repeats decrease TMPRSS2 expression.
- Histone demethylases are associated with the generation of constitutively active forms of AR variants.

**Estrogen receptors**

- Among *ACE2* regulatory regions, there are many estrogen receptor-binding motifs.
- No clear picture is emerging and the role (if any) of estrogen in coronavirus disease 19 (COVID-19) remains to be elucidated.
- Different polymorphisms have been described in both the *ESR1* and *ESR2* genes.
- *ESR1* and *ESR2* are downregulated by DNA methylation.
- Estrogen signaling is also tightly connected with post-translational histone modifications.
- The potential association of genetic and epigenetic alterations on *ESR1* and *ESR2* with SARS-CoV-2 infection, can only be hypothesized at present.

**Genetic classifier for SARS-CoV-2 risk group stratification**

- It can be predicted that a polygenic risk score for SARS-CoV-2 infectivity and COVID-19 disease severity will eventually be created.
- We propose a preliminary SARS-CoV-2 polygenic risk classifier discriminating individuals carrying a favorable for reduced infectivity genotypic combination and those who are genetically burdened to a stronger interaction of SARS-CoV-2 with host cell entry machinery and potentially to an increased-severity COVID-19 course.
- *ACE2* variations should be a core element of this polygenic risk classifier.
- *TMPRSS2*, *FURIN*, *ADAM-17* and *AR* gene polymorphisms may also have a role in the SARS-CoV-2 infectivity.
- This approach provides a dynamic scaffold based on current knowledge that can be constantly updated and adapted as novel, validated genetic and epigenetic data keep deriving.
- The described variants affecting ACE2-based pathway for SARS-CoV-2 entry would also have a role in the personalization of treatment with agents targeting the virus entry machinery into host cells.

**Initiatives on SARS-CoV-2 genetics**

- To identify individuals at unusually high or low risk, the genetic determinants of SARS-CoV-2 susceptibility, severity and outcomes should be studied.
- The *COVID-19 host genetics initiative* aims to organize analytical activities across studies to identify genetic determinants of COVID-19 susceptibility and severity.
- Genomics England, in partnership with the Genetics of Mortality in Critical Care consortium, aim to analyze the genomes of COVID-19 patients to try to understand how genes may affect individual reaction to the virus.
